# Risk of Zika virus transmission in the Euro-Mediterranean area and the added value of building preparedness to arboviral threats from a One Health perspective

**DOI:** 10.1186/s12889-016-3831-1

**Published:** 2016-12-03

**Authors:** Camille Escadafal, Lobna Gaayeb, Flavia Riccardo, Elisa Pérez-Ramírez, Marie Picard, Maria Grazia Dente, Jovita Fernández-Pinero, Jean-Claude Manuguerra, Miguel-Ángel Jiménez-Clavero, Silvia Declich, Kathleen Victoir, Vincent Robert

**Affiliations:** 1 Institut Pasteur, Paris, France; 2 Istituto Superiore di Sanità, National Centre for Epidemiology, Surveillance and Health Promotion, Rome, Italy; 3 Instituto Nacional de Investigación y Tecnología Agraria y Alimentaria, Centro de Investigación en Sanidad Animal (INIA-CISA), Madrid, Spain; 4 CIBER Epidemiología y Salud Pública (CIBERESP), Madrid, Spain; 5 Institut de Recherche pour le Développement, MIVEGEC Unit, IRD 224 - CNRS 5290 - UM, Montpellier, France

**Keywords:** MediLabSecure, Laboratory network, Euro-Mediterranean area, One Health, Arboviruses, Zika virus, *Aedes* mosquito, Preparedness, Capacity-building, Risk assessment

## Abstract

In the alarming context of risk of Zika virus (ZIKV) transmission in the Euro-Mediterranean area, there is a need to examine whether capacities to detect, diagnose and notify ZIKV infections in the region are in place and whether ongoing capacity-building initiatives are filling existing gaps.

The MediLabSecure network, created in 2014, comprises 55 laboratories of virology and medical entomology and 19 public health institutions in 19 countries in the Balkans, North-Africa, the Middle-East and the Black Sea regions. It aims to set up awareness, risk assessment, monitoring and control of emerging and re-emerging vector-borne viruses. We here examine the actions and strategies that MediLabSecure has been implementing and how they will contribute to the prevention and control of the ZIKV threat in the Euro-Mediterranean area.

Capacity-building for arbovirus diagnostics is a major objective of the project and follows a methodological rather than disease-driven approach. This enables the implementation of laboratory trainings on techniques that are common to several arboviruses, including ZIKV, and putting into action appropriate diagnostic tools in the target region.

Moreover, by its One Health approach and the interaction of its four sub-networks in human virology, animal virology, medical entomology and public health, MediLabSecure is fostering intersectoral collaboration, expertise and sharing of information. The resulting exchanges (methodological, communication and operational) across disciplines and across countries, dedicated research on intersectoral collaboration and increasing diagnostic capacities are providing new paths and tools to public health professionals to face emerging viral threats such as a ZIKV epidemic in the Euro-Mediterranean region.

## Background

Due to the link between Zika virus (ZIKV) infection and neurological disorders/neonatal malformations, the WHO acknowledged the ongoing ZIKV outbreak as a public health emergency of international concern (PHEIC) [1–4]. At least three factors are necessary to enable the onset of an outbreak of mosquito-borne viral infections in humans: (i) presence of the virus; (ii) susceptibility of human populations to the virus; (iii) presence of populations of anthropophilic vectors with suitable vectorial capacity [5]. Therefore, these three factors need to be considered while assessing the risk of a ZIKV outbreak occurring in the Euro-Mediterranean area. The presence of the virus is now demonstrated in the region as a corollary of globalization and increasing international travelers’ traffic [6–8]. In addition, the human population is assumed to be largely susceptible with the marginal exclusion of the few naturally immunized travelers infected in endemic/epidemic areas. Finally, as we will discuss in this paper, there is a high probability of the presence in this geographical region of vector populations that have demonstrated a suitable vectorial capacity to ZIKV.

Indeed, ZIKV has been isolated from a variety of mosquito species, mainly of the Aedini tribe (among which the subgenera *Stegomyia*, *Diceromyia* and *Aedimorphus* of the genus *Aedes*) [9, 10], indicating a mild specificity between the mosquito host and the virus. These findings strongly suggest that the *Stegomyia* mosquitoes*,* including the invasive tiger mosquito *Aedes albopictus* that is spreading in the Euro-Mediterranean and Black Sea area [11, 12], comprises vector populations with a sufficiently high vectorial capacity to allow ZIKV transmission.

Considering this alarming context, the ECDC recently issued a ZIKV risk assessment for continental Europe, estimating an increasing number of travel-related reported ZIKV infections, and indicating a risk of transmission from imported cases by the *Aedes albopictus* mosquito species [13], now established in many parts of the European Union (EU), primarily around the Mediterranean [14]. This finding raises the issue of whether capacities to detect, diagnose and notify ZIKV infections in the region are in place and whether ongoing capacity-building initiatives are filling existing gaps.

**Fig. 1:**
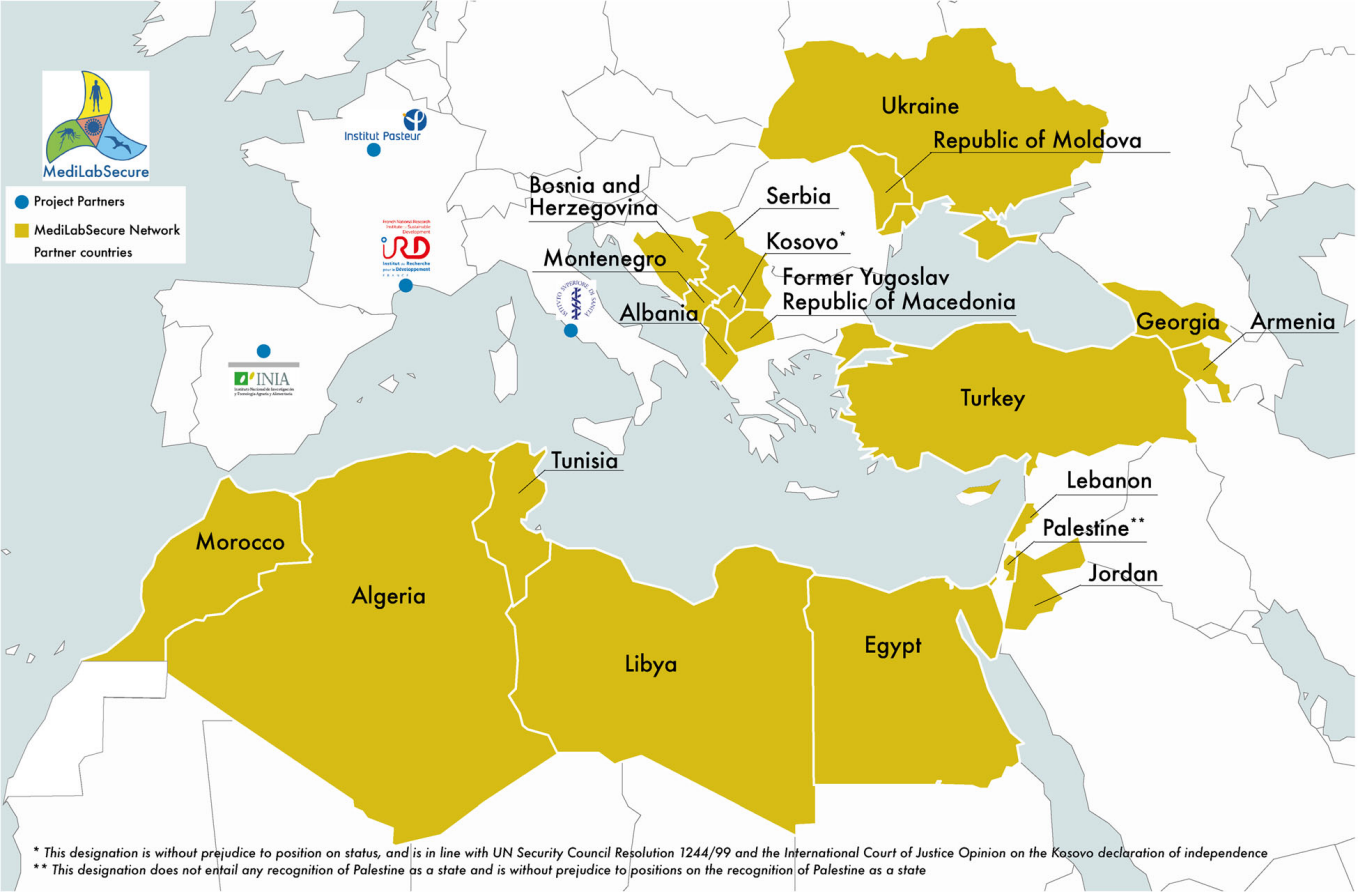
MediLabSecure network partner countries (*n* = 19) and project partners : Institut Pasteur, Paris, France (project coordination and human virology), National Centre for Epidemiology, Surveillance and Health Promotion, Italy (public health), Instituto Nacional de Investigación y Tecnologia Agraria y Alimentaria, Centro de Investigación en Sanidad Animal (INIA-CISA), Madrid, Spain (animal virology), Institut de Recherche pour le Développement, Montpellier, France (medical entomology). Figure 1 is produced by the authors of the present manuscript

In order to enhance preparedness to common health threats and biosafety risks, the EU supported the creation of the MediLabSecure network of human, veterinary and medical entomology laboratories and public health institutions which aims to support target countries to set up awareness, risk assessment, monitoring and control of emerging and vector-borne viruses of concern in the region. This network was created in 2014 as a continuation of a prior network developed by the EpiSouth and EpiSouth Plus Projects [15, 16] on the basis of the common needs and challenges shared by the countries bordering the Mediterranean Basin in the field of surveillance and control of infectious diseases and other threats to health. The strategies adopted by the EpiSouth Network were positively assessed by both internal and external evaluators during its implementation [17, 18] and its added value has been internationally recognized and rewarded with the 2014 European Health Award as “the Mediterranean project to counteract cross-border threats to health” [19]. Today, the MedilabSecure Network comprises 55 laboratories and 19 public health institutions/Ministries of Health in 19 non-EU countries in the Mediterranean and Black Sea regions (Fig. 1).

This One Health project rotates around the interaction of four sub-networks: one for human health, one for animal health, one for medical entomology and one for public health. This approach targets viral threats that can have a great impact on human and/or animal health.

The purpose of this correspondence is to present the actions and strategies that MediLabSecure is implementing and how they can now contribute to the prevention and control of the ZIKV threat. We aim to highlight how, although established prior to the current crisis, MediLabSecure has been implementing a number of WHO recommendations included in the ZIKV PHEIC Declaration [4] under a One Health perspective.

## Capacity-building activities impacting Zika virus preparedness

The overall MediLabSecure objectives are: (i) identify the needs of the different target laboratories by assessment tools, (ii) design and implement tailored training sessions accordingly, (iii) complement with expert visits and quality assessments and (iv) strengthen intersectoral collaborations between the human, animal and entomological sectors involved in vector-borne diseases surveillance. This objective is addressed by holding multidisciplinary “One Health” activities such as joint meetings, technical transversal workshops, expert visits where representatives of the four networks interact at national and international levels to foster mutual cooperation and intersectoral risk assessment exercises and tailored research to explore how this intersectoral collaboration is implemented within partner countries.

To date, the MediLabSecure **human virology sub-network**, under the leadership of the Institut Pasteur in Paris, conducted three workshops on scientific knowledge and technical skills (real-time RT-PCR and serology by ELISA) for the detection of arbovirus infections. While specific diagnostic capacity for ZIKV in terms of reagents and reference protocols was not initially an aim of this project, the MediLabSecure human virology sub-network reacted to this new public health context and, by January 2016, ZIKV real-time RT-PCR protocols were shared and required reagents including positive controls were distributed within the network. Information regarding the availability and performance of diagnostic tools and reference protocols as well as accurate testing algorithms are shared and regularly updated.

The MediLabSecure **animal virology sub-network**, under the leadership of the Animal Health Research Centre (INIA-CISA, Spain), is working to enhance preparedness of veterinary laboratories to face health threats caused by emerging viral pathogens, most of them zoonotic in nature. Technical workshops, experts’ exchanges, scientific visits and external quality assessment exercises are performed to improve the capacities of the laboratories to diagnose zoonotic viral infections in animals, with special focus on arboviruses. Finally, the overall objectives are completed by promoting continuous contact and exchange of information not only between veterinary laboratories from the different participating countries, but also within the different sub-networks, in-line with the adopted “One Health” perspective.

Although ZIKV is not regarded as a zoonotic agent, there are still a number of uncertainties around its host range and life cycle. For instance, there is evidence that bats could play a role in ZIKV infectious cycle [20]. More studies are needed to clarify the involvement of animal hosts on ZIKV epidemiology and its possible role in animal disease. In case new studies would reveal the involvement of other vertebrates in ZIKV infectious cycle, appropriate detection and control tools would be rapidly disseminated and implemented through the animal virology network based on previously shared protocols and techniques taught during the workshops.

The MediLabSecure **medical entomology sub-network**, under the leadership of the Research and Development Institute (IRD, France), focuses mainly on mosquitoes identified as present threats for arbovirus transmission but also with potential risk of emergence in the Mediterranean and Black Sea regions. Expert visits, and training modules have been implemented and mosquito identification tools have been devised. Three one-week training sessions have enabled at least two entomologists from each of the 19 laboratory members to increase practical competencies concerning mosquito vectors of arboviruses (field sampling, species determination, surveillance systems) and to enhance regional cooperation.

In view of strengthening integration across disciplines for surveillance of vector-borne diseases, the MediLabSecure **public health sub-network** designed a framework to define, study and promote intersectoral integration of surveillance from a One Health perspective through a systematic literature review, a survey involving all the sub-networks in all 19 countries [21] and the MediLabSecure Situation Analysis Study. Preliminary findings were consolidated during a public health workshop where 73 representatives of all the described sub-networks were engaged in an exercise on intersectoral WNV risk assessment and SWOT analysis. While not specifically addressing ZIKV surveillance, this work has involved the same professionals currently engaged in the detection of ZIKV infections from a virological, entomological and public health perspective and created a unique opportunity for team building, exchange of expertise and information across the different sectors and countries.

## Conclusion

The current ZIKV emergence has highlighted the need to conduct comprehensive risk assessments, establish effective monitoring programs, develop preparedness activities and implement adequate control measures with regard to arboviral threats in the Euro-Mediterranean area. Thanks to its One Health framework integrating four sub-networks involved in the monitoring, prevention and control of arboviral diseases from different perspectives, MediLabSecure is fostering collaboration, networking and timely sharing of information, across disciplines and across countries. In fact, as also discussed by other authors [22], the desired impact of the One Health approach expected through intersectoral integration can only be achieved if also the capacities of each involved sector are sufficiently strong and developed. MedilabSecure is working with a comprehensive strategy addressing both the capacity of the single sector and the intersectoral integration.

The resulting exchanges and capacity-building are now facilitating the step-up of intersectoral regional capacities in the face of emerging viral threats in a methodological rather than disease driven approach. Therefore, after having developed the basic methodological skills across the region, the network is now in the position to fill gaps in ZIKV detection capacity, by putting into action harmonized diagnostic tools and algorithms, vector sampling and identification methods and control measures, within a conducive intersectoral framework which constitutes an asset towards preparedness to any present or emerging threats to human and/or animal health.

## Abbreviations

ELISA: Enzyme-Linked Immunosorbent Assay
EU: European Union
PHEIC: Public health emergency of international concern
RT-PCR: Reverse transcription polymerase chain reaction
SWOT: Strengths, weaknesses, opportunities, threats
WHO: World Health Organization
WNV: West Nile virus
ZIKV: Zika virus
